# Prediction of Drug-Induced Liver Toxicity Using SVM and Optimal Descriptor Sets

**DOI:** 10.3390/ijms22158073

**Published:** 2021-07-28

**Authors:** Keerthana Jaganathan, Hilal Tayara, Kil To Chong

**Affiliations:** 1 Department of Electronics and Information Engineering, Jeonbuk National University, Jeonju 54896, Korea; keerthanairtt@gmail.com; 2 School of International Engineering and Science, Jeonbuk National University, Jeonju 54896, Korea; 3 Advanced Electronics and Information Research Center, Jeonbuk National University, Jeonju 54896, Korea

**Keywords:** drug-induced liver toxicity, feature selection, support vector machine, prediction, molecular descriptors

## Abstract

Drug-induced liver toxicity is one of the significant safety challenges for the patient’s health and the pharmaceutical industry. It causes termination of drug candidates in clinical trials and also the retractions of approved drugs from the market. Thus, it is essential to identify hepatotoxic compounds in the initial stages of drug development process. The purpose of this study is to construct quantitative structure activity relationship models using machine learning algorithms and systematical feature selection methods for molecular descriptor sets. The models were built from a large and diverse set of 1253 drug compounds and were validated internally with 10-fold cross-validation. In this study, we applied a variety of feature selection techniques to extract the optimal subset of descriptors as modeling features to improve the prediction performance. Experimental results suggested that the support vector machine-based classifier had achieved a better classification accuracy with reduced molecular descriptors. The final optimal model provides an accuracy of 0.811, a sensitivity of 0.840, a specificity of 0.783 and Mathew’s correlation coefficient of 0.623 with an internal validation set. Furthermore, this model outperformed the prior studies while evaluated in both the internal and external test sets. The utilization of distinct optimal molecular descriptors as modeling features produce an in silico model with a superior performance.

## 1. Introduction

The liver is an indispensable organ of the body due to its crucial contribution in metabolizing xenobiotics [1]. Drug-induced liver toxicity is one of the primary reasons for drug failure in clinical cases and also leads to termination of approved drugs from the market. Most commonly, drugs, herbals and other dietary products are responsible for the uncertain adverse liver injury [2,3,4,5]. The idiosyncratic behavior of the drugs not only caused by the dose level prescribed but also depends on the patient’s metabolic, genetic and immunological factors [6]. Due to the unpredictable adverse hepatic effects on patient’s health, drug-induced liver injury (DILI) risk assessment has become the most important concern for safe drug development [7,8,9,10]. Hence, it is required to concentrate more on identifying the potential hepatotoxic compounds in advance.

Animal studies for predicting DILI concerns in the preclinical assessment are not reliable, as it provides a low correlation results in clinical trials and also in post-marketing treatment [11,12]. In vitro and in vivo experiments for detecting DILI of large number of substances are time-consuming and expensive. Additionally, most of the compounds induce peculiar toxicity effects in human liver which cannot be discovered by the regulatory system for new drugs [13,14,15,16]. To address the limitations of experimental approaches, predictive computational modeling is taken into consideration for evaluating the DILI risk of drug candidates. Moreover, computational studies are reasonably cheaper, allows rapid prediction in virtual screening of huge compounds and evade ethical challenges linked to animal methods [17,18].

In recent years, computational predictive modeling approaches have been recognized as an alternative by many research groups. Despite various data types, for example, chemical structure and gene expression data, more number of computational models using the molecular structure of the compounds have been reported [19,20,21,22]. In particular, in silico studies are beneficial for filtering out molecular structures causing hepatotoxicity in the early stages of drug discovery. However, expert-based models using structural alerts are not successful predictors as they are defined according to experts’ experience and knowledge about the drug toxicity mechanisms [23,24,25]. So, various machine learning algorithms based on statistical Quantitative Structure Activity Relationship (QSAR) models have been developed by using the molecular structure features with the known hepatotoxicity endpoint datasets. Ekins et al. developed a Bayesian model using extended connectivity molecular fingerprints and interpretable descriptors based on a training set composed of 295 compounds and a test set of 237 compounds. This Bayesian model had a prediction accuracy of about 60% in external validation data [26]. Zhang et al. presented a naive Bayes classifier, which yielded an accuracy of 72.6% for the external test set [27]. Although many machine learning-based statistical models have been reported with sufficiently high accuracy, these models suffered from either imbalanced or small datasets with unsatisfactory prediction performances [28,29,30]. Mulliner et al. published Support Vector Machine (SVM) models combined with a genetic algorithm trained on a large dataset of 3712 compounds related to human and animal liver toxicity data [31]. Ai et al. reported an ensemble learning model using molecular fingerprints based on 1241 diverse compounds [32]. Recently, He et al. built a large and chemically diverse balanced training set of 1254 unique compounds as a result of system literature retrieval and constructed an ensemble model by integrating eight base classifiers to enhance prediction performance using molecular descriptors given by Marvin [33]. They achieved an average accuracy (ACC) of 0.783, sensitivity (SEN) of 0.818 and specificity (SPE) of 0.748 within a 10-fold cross-validation. Altogether, the prediction performance of the proposed models are not satisfactory, and there is substantial room for enhancing drug-induced liver toxicity predictions.

**Figure 1:**
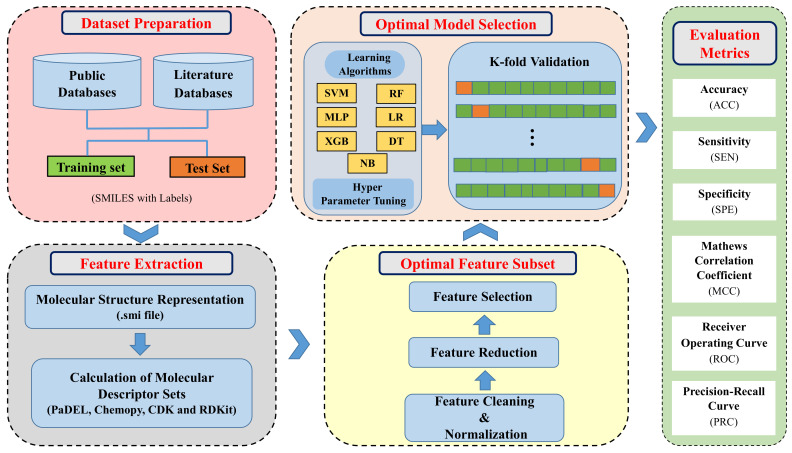
Illustration of the overall workflow.

In this present study, we propose a drug-induced liver toxicity prediction model using an SVM classifier with optimal subset of numerically represented molecular structure features. We worked on a variety of machine learning methods and feature selection techniques to improve the liver toxicity prediction performance using molecular descriptors. We computed different molecular descriptor sets from compounds’ Simplified Molecular Input Entry System (SMILES) format using various open software such as PaDEL, Chemopy, CDK and RDKit. We employed feature reduction techniques to remove redundant and irrelevant features from high dimensional molecular descriptor sets. Then, we applied feature selection techniques F-score algorithm for feature ranking followed by SVM linear kernel-based Recursive Feature Elimination with Cross-Validation (RFECV) method to select the optimal subset of features. Initially, we analyzed the performance of the SVM classifier with the optimal features of each individual molecular descriptor sets. Next, we investigated the prediction performance with different combinations of individual descriptor sets. Finally, the combination of all descriptor sets was used to build binary machine learning classifiers. The SVM-based classifier with reduced molecular descriptors showed improved prediction performance within 10-fold cross-validation and external validation set compared to the recently reported prior study. The overall workflow of the proposed model is shown in Figure 1.

## 2. Materials and Methods

### 2.1. Datasets

We obtained the training dataset compounds to develop a drug-induced liver toxicity prediction model from previously published work [33]. He et al. constructed a training dataset by integrating most of the data from publicly available datasets, i.e., DILIrank [34], LiverTox [35], and LTKB [36], and also performed an extensive study from the PubMed database and various scientific publications [37,38] to retrieve new hepatotoxic and hepaprotective compounds. Furthermore, a crucial data filtering procedure was performed to make a large scale and chemically diverse training set of 1254 compounds. The Simplified Molecular Input Line Entry System (SMILES) for each compound was acquired from the PubChem compound database [39]. We excluded a compound from the previous study because it may create an outlier as most of the compounds have SMILES sequence lengths of less than 150. Finally, our training set has 1253 compounds, consisting of 636 hepatotoxic and 617 non-hepatotoxic compounds. We collected drug compounds for the test dataset from the literature [33,40]. After eliminating duplicate and structurally similar compounds, we randomly selected 208 drug compounds, consisting of 94 hepatotoxic and 114 non-hepatotoxic compounds.The training and test datasets used in this study can be found in the supplementary file (Tables S1 and S2).

### 2.2. Molecular Descriptors

**Figure 2:**
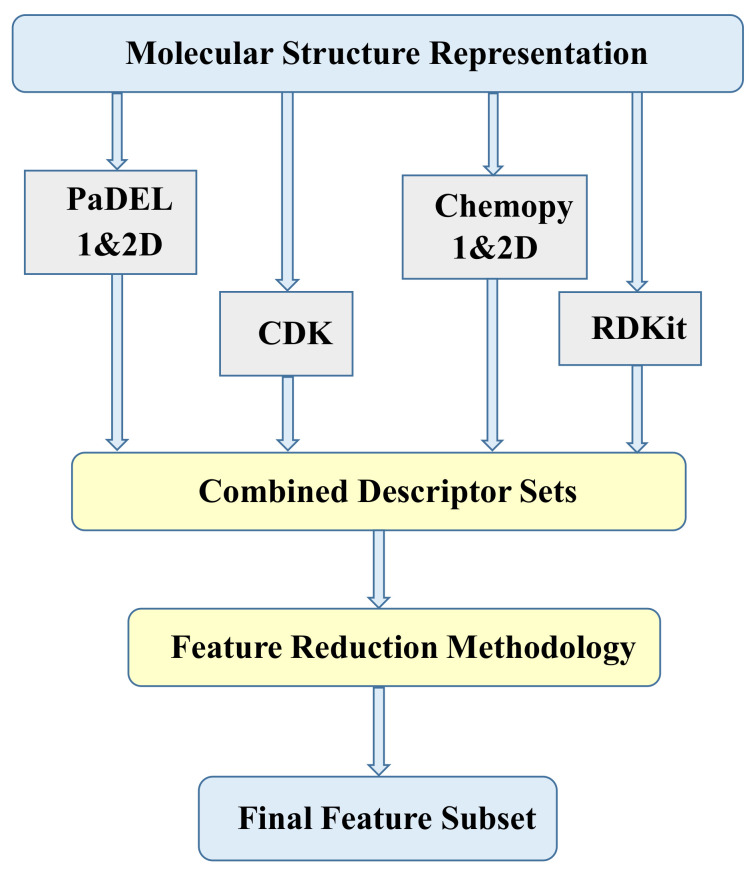
Combining individual descriptor sets.

Molecular descriptors are commonly utilized to quantitatively represent molecular characteristics for drug compounds [41]. We can compute numerous descriptors from the SMILES string format through various open source packages [42,43,44,45]. In this study, we calculated four sets of descriptors (CDK, Chemopy, PaDEL and RDKit) using an integrated publicly available web-based platform ChemDes [46]. We used individual descriptor sets and their combinations as shown in Figure 2. The individual descriptor set count is shown in Table 1. In total, 2648 descriptors were computed. In combined descriptor sets, the redundant descriptors which were calculated by more than one software were eliminated in the data preprocessing phase as discussed in upcoming section.

### 2.3. Data Preprocessing and Feature Selection

**Figure 3:**
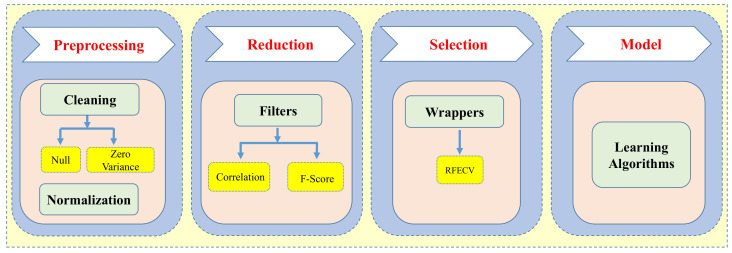
Illustration of the phases to develop final descriptors subset.

Data preprocessing is an essential step in machine learning modeling as it improves the quality of the data and impacts the learning capability of the model. The descriptor preprocessing, reduction and selection methodology is shown in Figure 3.

The main purpose of data cleaning is to identify and remove the noisy data by dropping the missing and identical value features. A variance threshold algorithm was applied to remove the zero variant features, i.e., the features with the same value in all drug compounds. The selection of most important subset of features is the challenging optimization step in machine learning-based model development. Feature selection techniques reduce the computational cost and complexity of the model. There are several feature selection techniques to select the best molecular descriptor subset for training the model [47]. We utilized the feature selection algorithms implemented by open source machine learning library Scikit-learn [48] in Python. Filter-based selection methods are faster and generally used in the case of the high dimensional features. In filtering, the selection of features i performed without considering the predictive model. The filter-based linear correlation method was used to eliminate the redundant and irrelevant features by using the Pearson correlation coefficient [49]. Molecular descriptors having a mutual correlation of more than 0.9 have been reduced by dropping one of the highly correlated features. The F-score algorithm was implemented to rank all the features according to the feature importance score. The feature importance score was calculated based on the correlation value of each feature with the target label and not considering the mutual information among the features [50].

In addition to filter methods, wrapper methods were proposed to search for the best performing subset of features by iterative training of a supervised learning estimator. Though wrapper-based selection methods are computationally expensive, it avoids over-fitting and improves the learning accuracy of the predictive model. We applied the Recursive Feature Elimination and Cross Validation (RFECV) technique to select the high ranked features by training the SVM linear classifier while recursively eliminating the low importance features [51,52,53,54]. The optimal feature subset of 155 molecular descriptors was selected after eliminating 5% of less important molecular descriptors in each iteration using 10-fold CV method. The final optimized subset selected from the training set and external test set was used for model development, internal validation and external validation, respectively.

### 2.4. Model Building and Optimization

Machine learning models can be used to predict hepatotoxicity given the molecular descriptor of a compound as input. We mainly focused on the following machine learning algorithms to develop binary classification models, among several methods that have been applied in QSAR modeling: Support Vector Machine (SVM), Multi-Layer Perceptron (MLP), Logistic Regression (LR), Random Forest (RF), XG Boosting (XGB), K-Nearest Neighbors (KNN), Naive Bayes (NB) and Decision Tree (DT) classifier [27,32,33,53,55,56]. These robust algorithms are highly efficient and can accommodate numerous features. We implemented these machine learning algorithms by using the widely used library Scikit-learn in Python [48]. The machine learning algorithms’ performances can be effectively improved by tuning their parameter values. The hyper-parameters of the models were optimized by the grid search method with cross-validation over a parameter grid. We trained the optimized algorithms with the best selected molecular descriptors and known hepatotoxicity labels. In this study, an SVM-based binary classifier was mainly used for performance comparison.

#### Support Vector Machine

SVM is a powerful supervised learning method and widely used for solving classification problems. The algorithm performs the classification by identifying the optimal hyper-plane using several kernel functions that discriminate between the positive and negative class molecules in a high dimensional space. In this study, we used the most popular radial basis function (RBF) as the kernel function which showed better performance than the others (linear, sigmoid and polynomial). In addition, we optimized the penalty parameter C and the kernel coefficient Gamma of the RBF kernel through the grid search method with cross-validation. The regularization parameter C controls the trade-off between the smooth decision boundaries and correct classification [57]. The higher values of kernel width parameter Gamma denotes an exact fit as per the training dataset and causes an over-fitting problem. The optimal values of C and Gamma used in this study were 100 and 0.01, respectively.

### 2.5. Model Training and Validation

In this study, the reliability and quality of the proposed model was evaluated by performing external validation in addition to 10-fold cross-validation (CV). In the CV method, the training dataset was randomly divided into 10 subsets. The optimized model was trained with nine subsets and the remaining one subset as a internal validation set. The training and validation procedure was repeated ten times with different training subsets and internal validation sets, respectively. Finally, the performance of the binary classifier was calculated by averaging the results of the 10 corresponding internal validation sets.

### 2.6. Performance Evaluation Metrics

To assess the predictive ability of the proposed model, we employed several statistical metrics, including accuracy (*ACC*), the overall prediction accuracy; sensitivity (*SEN*), the prediction accuracy of hepatotoxic compounds; specificity (*SPE*), the prediction accuracy of non-hepatotoxic compounds; Matthew’s correlation coefficient (*MCC*); and *F*1-*Score*, which are mathematically defined as follows:

(1) $$ACC=\frac{TP+TN}{TP+TN+FN+FP}$$

(2) $$SPE=\frac{TN}{TN+FP}$$

(3) $$SEN=\frac{TP}{TP+FN}$$

(4) $$MCC=\frac{\left(TP*TN)-(FP*FN\right)}{\sqrt{\left(TP+FP)(TP+FN)(TN+FP)(TN+FN\right)}}$$

(5) $$F1-Score=\frac{2*TP}{(2*TP)+FN+FP}$$

(6) $$RndACC=\frac{\left(TP+FN)*(TP+FP)+(TN+FP)*(TN+FN\right)}{N^{2}}$$

(7) ΔACC=100∗(ACC−RndACC)(%)

where true positive (*TP*) denotes the number of hepatotoxic molecules that are predicted correctly, true negative (*TN*) indicates the number of non-hepatotoxic molecules that are predicted correctly, false positive (*FP*) is the count of non-hepatotoxic compounds that are incorrectly predicted as hepatotoxic compounds, false negative (*FN*) is the count of hepatotoxic compounds that are incorrectly predicted as non-hepatotoxic compounds. The MCC is used to measure the balanced classification performance, the coefficient value 1 indicates perfect classification and −1 represents perfect misclassification [58]. The statistical parameter F1-Score is calculated for estimating the quality of binary classification models using an imbalanced dataset. The random accuracy (*RndACC*) and its difference with real accuracy (Δ*ACC* in %) can be estimated to rank the predictive quality of the QSAR models [59]. The receiver operating curves (ROCs) and the precision recall curves (PRCs) were plotted to summarize the binary classification performance. Additionally, we calculated the area under the ROC curve (AUC-ROC) and the area under the PRC curve (AUC-PRC) for classifier comparisons.

## 3. Results and Discussion

### 3.1. Data Analysis

**Figure 4:**
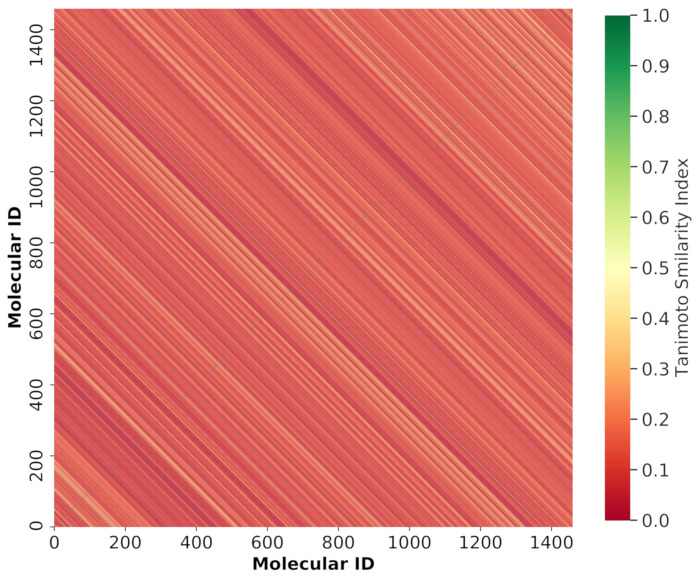
Heat map showing the molecular similarity of the molecules used in the entire dataset plotted by Tanimoto similarity index calculated using Morgan Fingerprints. The *x*-axis and *y*-axis represent the number of molecules used in the whole dataset.

**Figure 5:**
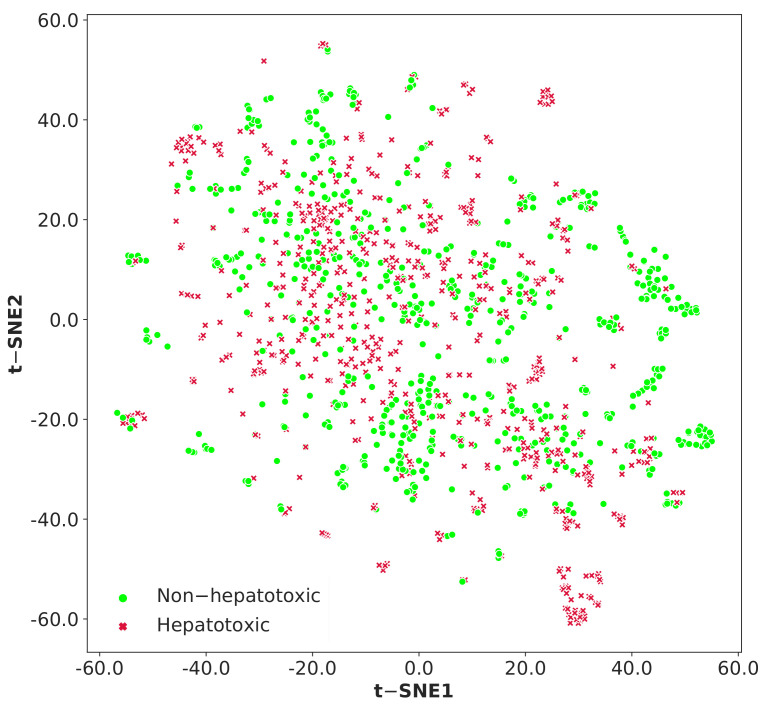
Visualizing the chemical space coverage using t-distributed stochastic neighbor embedding (t-SNE) on PCA output with 100 principal components (accounts for 56.76% of the overall variance) of the entire dataset (training and test datasets). Red x markers represent the hepatotoxic compounds and the green circle markers represent the non-hepatotoxic compounds.

**Figure 6:**
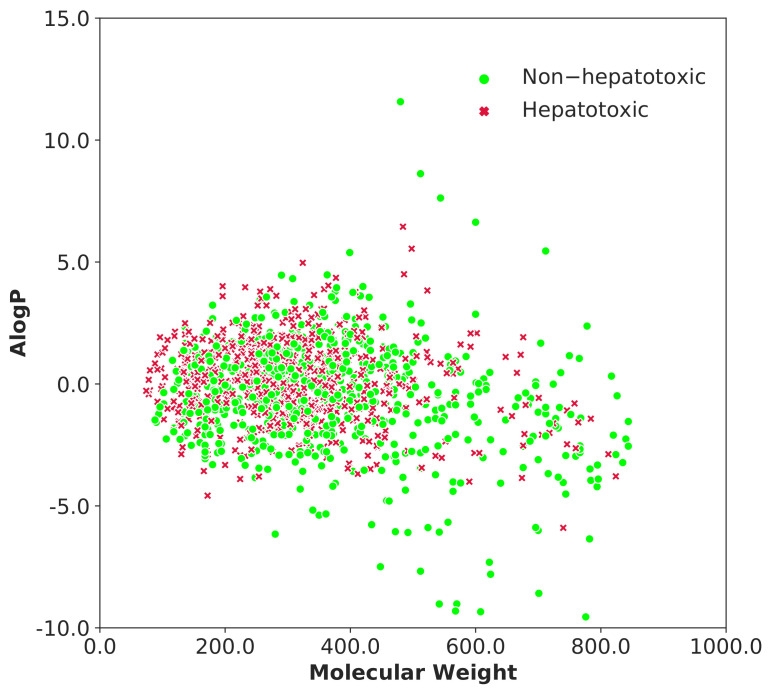
Chemical space defined by molecular weight and AlogP of the entire dataset (training and test datasets). Red x markers represent the hepatotoxic compounds and the green circle markers represent the non-hepatotoxic compounds.

To estimate the chemical diversity of the dataset used in this study, we calculated the Tanimotto similarity index [60] based on Morgan Fingerprint with radius 2. The majority of the compounds in the training and test sets had similarity indices in the range below 0.30 and the mean value was only 0.0921. These results suggest that the chemical structures used in our dataset were diverse. We plotted the heat map corresponding to the Tanimotto similarity index of molecules from the entire dataset (Figure 4).

t-distributed stochastic neighbor embedding (t-SNE) is a non-linear technique for dimensionality reduction and it is used to create graphical representation of the chemical space covered by the set of molecules [61]. It is recommended to reduce the number of dimensions before performing the t-SNE algorithm, which will speed up the computation and suppress some noise. In this study, principal component analysis (PCA) was performed on 2048 bit Morgan fingerprints to obtain 100 principal components, which represent 56.76% of the overall variance in the data. Figure 5 represents the chemical space visualization of the entire dataset using t-SNE algorithm. Furthermore, we explored the chemical space of the whole dataset using molecular weight and AlogP (octanol/water partition coefficient) as demonstrated in Figure 6. The molecular weight values varied from 74 to 843.88 and AlogP values ranged from −7.88 to 15.61. The scatter diagram distributions (Figure 5 and Figure 6) illustrate that the hepatotoxic and non-hepatotoxic compounds shared the same chemical space.

### 3.2. Performance of Models Using Cross-Validation

Various machine learning methods were used to build prediction models based on molecular descriptor subsets. Initially, we worked on individual descriptor sets and evaluated their performances with few machine learning models. Then, we made different combinations of molecular descriptor subsets and found a good combination of the descriptor set that produced the best prediction results for the validation data. The best prediction model was built based on the selected combination of the individual descriptor sets.

#### 3.2.1. Experiments with Individual Descriptor Sets

We employed the experimental workflow approach on the four individual descriptor sets such as CDK, Chemopy, PaDEL and RDKit. At first, empty valued and zero variant features were removed from the original molecular descriptor set. After applying the feature importance score-based ranking and RFECV techniques, we selected an optimal subset of molecular descriptors from each set for model training and validation. The SVM-based classification model was used to compare the 10-fold cross-validation performance of the individual descriptor sets.

Table 2 shows the selected number of descriptors from each set and their prediction performance results. It is evident that the PaDEL descriptor set performs better in terms of accuracy (ACC), Sensitivity (SEN) and Mathews Correlation Coefficient (MCC) than the other individual descriptor sets.

#### 3.2.2. Experiments with Combined Descriptor Sets

We examined various combinations of the individual descriptor sets to improve the model performance. Here, the similar experimental workflow, i.e., the preprocessing and feature selection methods, were also applied for the combined feature sets to obtain the low dimensional optimal descriptor subset. The PaDEL descriptor set was present in all the combination groups as it computes a large number of descriptors and showed better prediction performance compared to the other individual descriptor sets. The optimal number of descriptors and their SVM classifier prediction outcomes for different possible combinations are shown in Table 3.

From Table 3, it can be seen that every combined descriptor set showed improved prediction ACC and MCC compared to the best performing PaDEL descriptor set with 91 optimal descriptors. The number of descriptors for each group has been obtained as the result of optimizing the feature selection algorithms mentioned in the methods section. The combined group of three descriptor sets with 162 optimal features gave improved MCC over PaDEL-RDKit combination with 132 features. The combination of all the individual descriptor sets selected less than 6% of features after feature reduction and selection steps from the total number of 2648 original features. This combo showed an improved prediction in 10-fold cross-validation compared to all other combinations with respect to evaluation metrics ACC, SEN and MCC.

The details of 155 best selected descriptor subsets from combination of all the descriptor sets are given in the supplementary file (Table S3). Most of the selected features were from the PaDEL descriptor set and belong to auto-correlation, E-state and topological descriptors (Table 4). From Chemopy 1&2D, most of the descriptors are E-state and MOE (Molecular Operating Environment) descriptors. For CDK, topological and Kappa descriptors represented the major part. At last, all the selected RDKit descriptors are constitutional descriptors.

**Figure 7:**
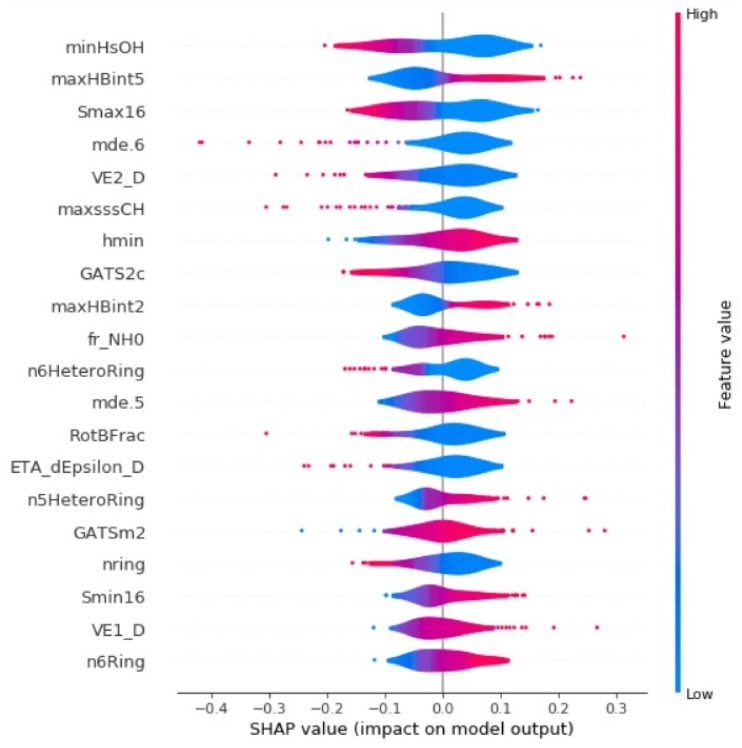
SHAP summary plot displays the distribution of top 20 important descriptors used for training the proposed model for hepatotoxicity prediction.

The Shapeley Additive Explanations (SHAP) technique is adopted to understand the most important descriptors and their contribution to the model prediction [62,63]. The SHAP technique is based on the game theory approach and was developed using Python. Figure 7 shows the summary plot of top 20 descriptors used for training the proposed SVM model. The SHAP summary plot indicates the relationship between the descriptor value and its impact on the model prediction. In the violin plot, the red color indicates the higher feature values and the blue color indicates the lower feature values. The descriptors are ordered based on their importance. The E-state descriptor “minHsOH” is the primary feature and it causes either a large positive or large negative in the model outcome and “maxHBint5” is the next most important descriptor.

#### 3.2.3. Comparison of SVM with Other Classifiers

**Figure 8:**
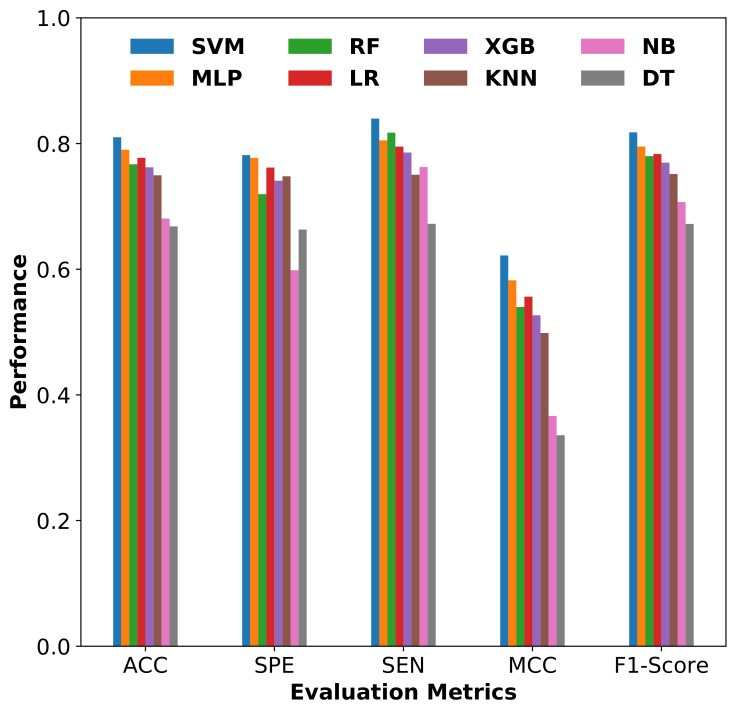
Performance comparison of various classifiers using 10-fold cross-validation.

We evaluated the performance of the SVM classifier built on the final selected optimal descriptors subset with other conventional supervised learning methods such as Multi-Layer Perceptron (MLP), Logistic Regression (LR), Random Forest (RF), XG Boosting (XGB), K-Nearest Neighbor (KNN), Naive Bayes (NB) and Decision Tree (DT) classifier. The performance evaluation metrics comparison of all classifiers using a 10-fold cross-validation is shown in Figure 8. The comparison results confirm that the SVM-based binary classification model is performing better for drug-induced liver toxicity prediction. Particularly, the accuracy of SVM is 14.2% more than DT, 13% more than NB, 6% more than KNN, 4.8% more than XGB, 4.3% more than RF, 3.3% more than LR, 2% more than MLP. The MCC value of the SVM-based model is above 0.6. In addition, the F1-score value of the proposed SVM-based model is above 0.8, which shows the quality of the binary classification model using imbalanced data.

The most probable random accuracy (RndACC) is calculated from confusion matrix values for all the models used for performance comparison and all the models reported in this study have a maximum random accuracy of value 0.5. The difference (ΔACC in %) between the real model accuracy (ACC) and the random accuracy was also calculated and used for ranking the models. The descending order ranking of the models based on this values is SVM, MLP, LR, RF, XGB, KNN, NB, DT classifier. The proposed SVM-based model has the highest accuracy difference value among all the models used for comparison. The confusion matrix values (TN, FP, FN & TP) and other evaluation parameter values (ACC, SPE, SEN, MCC, F1-Score, RndACC& ΔACC) of the 10-fold cross-validation for each model used in the comparison is provided in the supplementary file (Table S4).

**Figure 9:**
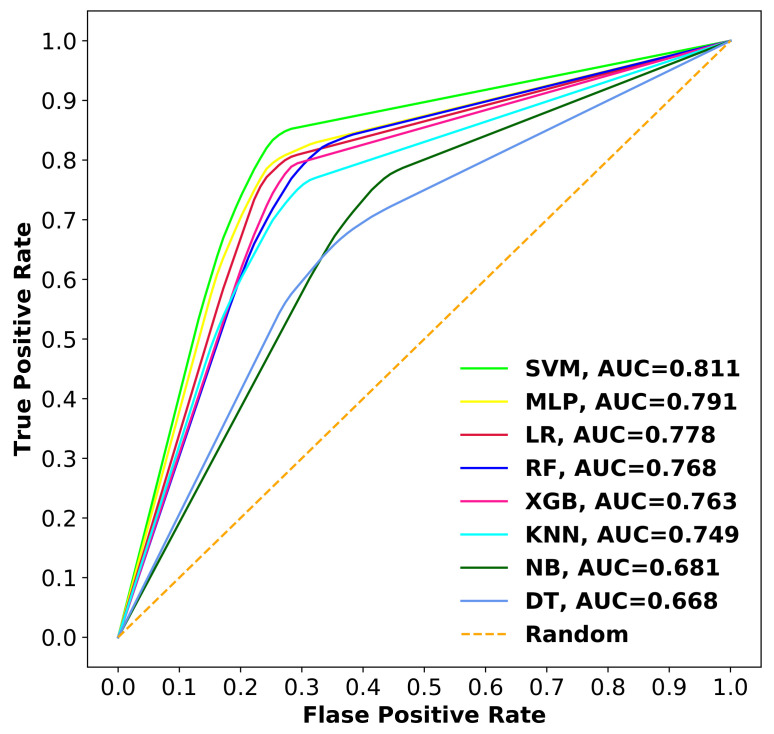
ROC comparison of different classifiers with corresponding AUC values using 10-fold cross-validation.

**Figure 10:**
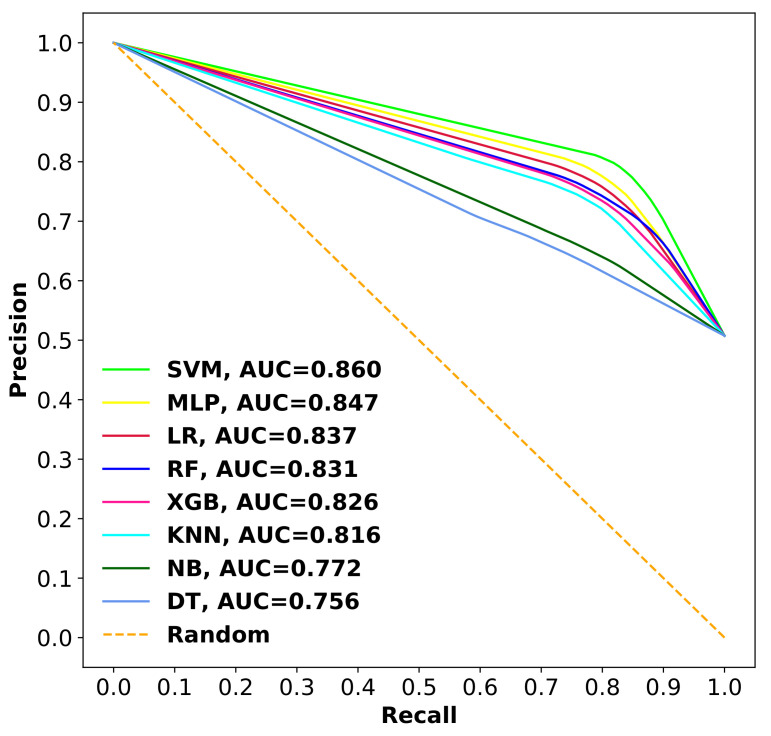
PRC comparison of different classifiers with corresponding AUC values using 10-fold cross-validation.

The comparison of receiver operating characteristic (ROC) curves and precision recall curves (PRCs) with area under the curve (AUC) values for all the models are shown in Figure 9 and Figure 10. As depicted from the results, SVM with the Radial Basis Function (RBF) kernel achieved AUC-ROC of 0.811 and AUC-PRC of 0.860, which are relatively better than all other methods.

### 3.3. Performance Comparison with Previous Work

Various QSAR models have been published for drug-induced liver toxicity prediction by using machine learning algorithms [27,28,29,31,32,37]. We only selected computational models that were cross-validated [27,28,32,33] for comparing with our proposed model. Although the proposed prediction model showed good performance in internal validation, it is required to perform the external validation to determine the robustness of the SVM-based model. In particular, the external validation dataset used in this study did not have any identical or high structural similarity compounds with the training dataset.

Table 5 shows that the results of cross-validation and external validation set comparison between proposed model with the previously published models. The accuracy of the proposed model is better than the other methods considered for comparison. The recently published ensemble model [33] yielded an ACC of 0.783, SPE of 0.748, and SEN of 0.818 with the 10-fold cross-validation. These results were improved with our proposed model, ACC by 2.8%, SPE by 3.5%, SEN by 2.2% and with a good MCC value of 0.623 (MCC was not given in the prior work). The SVM-based proposed model also achieved good performance compared to the ensemble model in external validation. Thus, the proposed SVM-based model is a promising hepatotoxicity predictor compared to the ensemble model.

## 4. Conclusions

Drug-induced liver toxicity estimation is one of the significant safety related challenges in the pharmaceutical industry. In this study, we focused on the prediction of liver toxicity based on computational models using a large and diverse dataset of 1253 unique compounds. We used a total of 2648 molecular descriptors calculated from four different descriptor sets as modelling features. Initially, null values and highly correlated features were dropped from the high dimensional feature space, and then feature selection techniques were applied to select the optimal subset of molecular descriptors for effective model training. Eight different supervised learning models were constructed and optimized with the best selected final features and their cross-validation prediction performance was analyzed. The SVM-based binary classification models utilizing less than 6% of the original features achieved improved performance compared to the other machine learning models. Moreover, the proposed model demonstrated better performance than the previous study in 10-fold cross-validation and external validation. It was observed from the comparison that the extended molecular descriptor feature space could improve the prediction performance. Meanwhile, the selection of discriminating model features is also a challenging task to obtain good prediction results. In the future, with great understanding of drug-induced liver toxicity mechanisms, we intend to investigate deep learning architectures using improved dataset considering biological data along with the chemical structure for improving the hepatotoxicity prediction. Additionally, a large-scale dataset with standard DILI definition and dose-level information will aid to build an efficient models for DILI assessment.

## Figures and Tables

**Table 1:** Details of the descriptor sets.

Descriptor Set	Descriptors Count
PaDEL 1&2D	1544
Chemopy 1&2D	633
CDK	275
RDKit	196
Total	2648

**Table 2:** The best performance of individual descriptor sets.

Descriptor Set	Selected No. of Descriptors	ACC	SPE	SEN	MCC
CDK	77	0.771	0.772	0.773	0.545
Chemopy	104	0.766	0.763	0.773	0.536
PaDEL	91	0.781	0.749	0.815	0.565
RDKit	86	0.752	0.729	0.777	0.507

**Table 3:** Performance details of best combination descriptor sets.

Best Combination Descriptor Sets	Optimal No. of Descriptors	ACC	SPE	SEN	MCC
PaDEL-RDKit	132	0.796	0.784	0.809	0.593
PaDEL-RDKit-CDK	162	0.804	0.796	0.813	0.609
PaDEL-RDKit-CDK-Chemopy	155	0.811	0.783	0.840	0.623

**Table 4:** Details of the selected optimal descriptor subset.

Descriptor Set	Descriptor Type	Total-Type	Total-Set	% of Selection
PaDEL 1&2D	Autocorrelation Descriptors	46	83	54
	E-state Descriptors	13		
	Topological Descriptors	11		
	Constitutional Descriptors	11		
	Others	2		
Chemopy 1&2D	MOE-type descriptors	11	37	24
	E-state Descriptors	11		
	Autocorrelation Descriptors	10		
	Others	5		
CDK	Topological Descriptors	17	28	18
	Kappa Descriptors	5		
	Autocorrelation Descriptors	3		
	Others	5		
RDKit	Constitutional descriptors	7	7	4

**Table 5:** Proposed model performance compared with the previously published literature.

Model Name	No. of Compounds	Test Method	ACC	SPE	SEN
Proposed Model	1253	10-fold CV	0.811	0.783	0.840
	208	External Validation	0.756	0.708	0.807
Ensemble Model [33]	1254	10-fold CV	0.783	0.748	0.818
	204	External Validation	0.730	0.658	0.773
Ensemble-Top5 [32]	1241	5-fold CV	0.711	0.603	0.799
SVM [27]	978	5-fold CV	0.797	0.585	0.948
	88	External Validation	0.750	0.379	0.932
RF [28]	996	10-fold CV	0.65	0.62	0.68
	966	External Validation	0.58	0.38	0.75

## Data Availability

No new data were generated in this study.

## Supplementary Materials

The following are available online at https://www.mdpi.com/article/10.3390/ijms22158073/s1.
